# Usefulness of Extra Virgin Olive Oil Minor Polar Compounds in the Management of Chronic Kidney Disease Patients

**DOI:** 10.3390/nu13020581

**Published:** 2021-02-10

**Authors:** Annalisa Noce, Giulia Marrone, Silvia Urciuoli, Francesca Di Daniele, Manuela Di Lauro, Anna Pietroboni Zaitseva, Nicola Di Daniele, Annalisa Romani

**Affiliations:** 1 UOC of Internal Medicine-Center of Hypertension and Nephrology Unit, Department of Systems Medicine, University of Rome Tor Vergata, Via Montpellier 1, 00133 Rome, Italy; giul.marr@gmail.com (G.M.); francesca.didaniele@gmail.com (F.D.D.); dilauromanuela@gmail.com (M.D.L.); annapietroboni@icloud.com (A.P.Z.); didaniele@med.uniroma2.it (N.D.D.); 2 PhD School of Applied Medical, Surgical Sciences, University of Rome Tor Vergata, Via Montpellier 1, 00133 Rome, Italy; 3 PHYTOLAB (Pharmaceutical, Cosmetic, Food Supplement, Technology and Analysis), DiSIA, University of Florence Via Ugo Schiff 6, Sesto Fiorentino, 50019 Florence, Italy; silvia.urciuoli@gmail.com

**Keywords:** extra virgin olive oil, polyphenols, antioxidant activity, chronic kidney disease, hydroxytyrosol, oleocanthal

## Abstract

Chronic kidney disease (CKD) is one of the most common chronic non-communicable degenerative diseases and it represents an important risk factor for cardiovascular morbidity and mortality. The Mediterranean diet, in which extra virgin olive oil (EVOO) is the main source of vegetal fats, represents a nutritional-diet regimen that is useful for the treatment of CKD and its comorbidities. We tested two different EVOOs, characterized by a high (Synergy) and medium (Luxolio) content of minor polar compounds (MPCs), detected by HPLC-DAD-MS analysis, in 40 nephropathic patients, at a dose of 40 mL/day for 9 weeks. We evaluated the effects of these two EVOOs on renal function, body composition, oxidative stress, and inflammatory state, after 9 weeks of EVOOs consumption (T1) and after 2 months of wash-out (T2). We observed an improvement of renal function biomarkers (estimated-glomerular filtration rate, albuminuria, azotemia, uric acid), lipid profile, oxidative stress, inflammatory parameters (erythrocyte sedimentation rate, C-reactive protein) and in body composition at T1. These healthy effects were greater and persisted over time after the wash-out period in Synergy patients. The high MPC EVOO content seems to exert an antioxidant and anti-inflammatory effect in nephropathic patients and these protective actions are maintained over time.

## 1. Introduction

Chronic kidney disease (CKD) represents one of the leading chronic non-communicable degenerative diseases in the world. CKD is associated with high morbidity and mortality, in addition, the costs of renal replacement therapy (RRT) have increased significantly from 1960 up to today [1]. In fact, the number of people who currently need RRT in the world exceeds 2.5 million and it is assumed that in 2030 this value will double and reach 5.4 million [2]. Furthermore, in many countries, especially in developing countries, there is little possibility of access to RRTs and it is hypothesized that 2.3–7 million adults will experience premature death due to the inability to undergo RRT [2]. Moreover, CKD is now a well-known independent risk factor for cardiovascular diseases (CVDs), specifically, starting from its earliest stages, an increase in cardiovascular morbidity and premature death is observed. This phenomenon can be explained by taking into consideration the cardiovascular (CV) risk factors of the uremic state [3]. In addition, CKD considerably worsens the quality of life of patients who are affected, therefore, it is fundamental to implement new therapeutic strategies aimed at slowing down of its progression and to counteract its comorbidities. Natural bioactive compounds represent a valid therapeutic alternative as they are characterized by a very low presence of side effects [4,5]. From this perspective, nutritional-diet therapy (DNT) plays a key role; in fact, it represents a fundamental part of treatment of CKD at all stages [6,7]. In the early stages of CKD, a “healthy diet” (HD) is recommended, and it is characterized by a high intake of vegetables, fruit, nuts, whole grains, legumes and fish, and low intake of saturated fats and sodium [8]. In fact, a recent meta-analysis revealed that HD was associated with lower mortality in CKD patients than in subjects who did not follow this practice [9]. A prospective population study “Singapore Chinese Health Study” highlighted the possible association between the intake of certain types of food and the incidence of end stage renal disease (ESRD). The authors demonstrated a strong correlation between the consumption of red meat and the risk of developing ESRD [10]. Furthermore, in our previous study we highlighted how the Mediterranean diet (MD) represents a nutritional-diet regimen valid for the treatment of the first stages of CKD, considerably reducing the CV risk associated with the disease [11,12]. This was confirmed by our subsequent study, which divided a population of nephropathic patients based on their genotype for the methylene tetrahydrofolate reductase (MTHFR) enzyme [13].

Among the typical products of an MD, a key food is represented by extra virgin olive oil (EVOO), which is its main source of vegetable fats. A total of 98–99% of the total weight of EVOO is made up by fatty acids, in particular, monounsaturated ones such as oleic acid, and a small percentage (1–2%) by minor polar compounds (MPCs). MPCs, especially of the phenolic variety, seem to be fundamental for the health effects exerted by EVOO. Among these, hydroxytyrosol, tyrosol, oleacin and oleocanthal are of particular importance [14,15]. In this regard, we tested two different EVOOs obtained from synergic-biodynamic agriculture, characterized by a high content of MPCs, evaluated by HPLC-DAD-MS analysis, in nephropathic patients. These EVOOs were administered to a group of CKD patients at a dose of 40 mL/day for 9 weeks in order to evaluate their effects on renal function, body composition, oxidative stress (OS) and inflammatory state. Furthermore, to confirm the potential results obtained, the patients underwent a wash-out period of two months, at the end of which the previously listed parameters were re-tested.

## 2. Material and Methods

### 2.1. Samples of EVOOs

EVOOs selected for this study were produced using biphasic pressing technology. The first oil selected was monocultivar Mignolo, produced in Tuscany (Vinci, Florence, Italy) in 2019 (Tuscany Region—European Partnership for Innovation in Agriculture Productivity and Sustainability 2014–2020—BioSynOL, “Oil and Legumes: biodynamic and synergistic crops for naturally fortified foods and innovative products for health and sport”). The second oil chosen was a mix of three cultivars of olives including Moraiolo (33%), Leccino (34%), and Mignolo (33%) produced in Tuscany (Vinci, Florence, Italy) in 2019. The monocultivar Mignolo oil was obtained from a biosynergy and biodynamic crop that was made with leguminous species capable of fixing important nutrients in the soil, that have positively influenced the quality of the olive tree and therefore of the oil produced. The two selected EVOOs were extracted and then analyzed by HPLC-DAD-MS for the evaluation of MPCs content, according to the protocol already described in another study conducted by our research group [16].

### 2.2. Patients and Methods

In the present clinical study, a group of 40 CKD patients (stages I–IV according to the National Kidney Foundation Kidney—Disease Outcomes Quality Initiative guidelines) [17] under conservative therapy, were enrolled at the UOC of Internal Medicine, Center for Hypertension and Nephrology Unit of the University Hospital Policlinico Tor Vergata (PTV), Rome. Subsequently, these patients were divided into 2 subgroups, matched by age, gender and body mass index (BMI): 20 patients assumed EVOO with medium MPCs (Luxolio, LUX) content and 20 patients assumed EVOO with a high MPCs content (Synergy, SYN).

**Figure 1:**
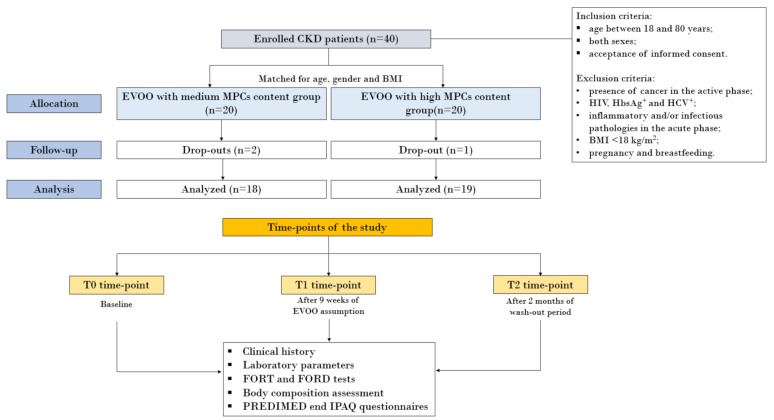
Flow-chart of the study. Abbreviations: BMI, body mass index; CKD, chronic kidney disease; EVOO, extra virgin olive oil; FORD, Free Oxygen Radical Defense; FORT, Free Oxygen Radical Test; IPAQ, International Physical Activity Questionnaire; MPCs, minor polar compounds; PREDIMED, Prevención con Dieta Mediterránea.

LUX and SYN were administered to nephropathic patients at a dose of 40 mL/day, for 9 weeks. In particular, the EVOOs were consumed raw, as a condiment for dishes. At baseline (T0), we collected the clinical history for each enrolled patient and we evaluated laboratory parameters, such as creatinine, azotemia, uric acid (UA), lipid profile, inflammatory and OS biomarkers, in addition to a body composition assessment. The same parameters were also examined at the two subsequent time-points: at time-point T1 (after 9 weeks of EVOO consumption) and at time-point T2 (after 2 months of wash-out period). The flow-chart of the study design is illustrated in Figure 1.

The study protocol was declared as being compliant with the Helsinki declaration by the PTV Independent Ethics Committee. All enrolled subjects signed an informed consent at the point of enrollment.

The inclusion criteria were individuals aged between 18 and 80 years, both sexes after acceptance of informed consent. The exclusion criteria were presence of cancer in the active phase, HIV, HbsAg^+^, HCV^+^, inflammatory and/or infectious pathologies in the acute phase; BMI < 18 kg/m^2^; pregnancy and breastfeeding. During the period of study, the patients did not undergo therapeutic changes.

All patients followed an Italian standard Mediterranean diet with a controlled protein intake according to CKD stage [18,19]. All patients were instructed to maintain a water intake between 1000–1500 mL/day so as to guarantee a neutral water balance.

### 2.3. Questionnaires

**Figure 2:**
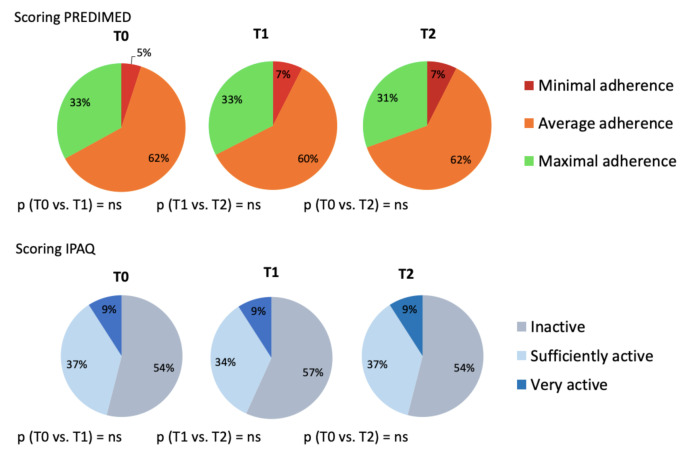
PREDIMED and IPAQ questionnaires. Abbreviations: IPAQ, International Physical Activity Questionnaire; PREDIMED, Prevención con Dieta Mediterránea.

Two questionnaires were administered to all nephropathic patients with the aim to exclude potential biases due to changes in eating habits or physical exercise during the study. PREDIMED (Prevención con Dieta Mediterránea) and IPAQ (International Physical Activity Questionnaire) questionnaires were performed at all time-points of the study (T0, T1, T2). The first assesses the degree of adherence to the Mediterranean diet [20] and the second evaluates the degree of weekly physical activity [21]. The PREDIMED and IPAQ questionnaires did not show any significant difference in the eating habits and lifestyle of the enrolled patients, for the entire study period (Figure 2).

In addition, to assess the polyphenols intake from other foods, we recorded a 24 h dietary recall at all time-points of the study, highlighting homogeneous polyphenols consumption between the patients, at each time point of the study [22].

### 2.4. Body Composition Assessment

Anthropometric parameters of all CKD patients were registered according to standard methods [23]. Body weight (kg) was measured to the nearest 0.01 kg, using a balance scale (Seca 711, Hamburg, Germany). Height (m) was measured using a stadiometer to the nearest 0.1 cm (Seca 220, Hamburg, Germany). BMI was calculated as body weight divided by height squared (kg/m^2^).

For the assessment of body composition, all nephropathic patients underwent bioelectrical impedance analysis (BIA). Resistance, reactance, impedance, and phase angle at 50 KHz frequency were measured using a BIA 101S instruments (Akern/RIL System-Florence). For evaluation of body composition, we considered the following parameters: total body water (TBW), intracellular water (ICW), extracellular water (ECW), body cell mass index (BCMI), fat free mass (FFM), fat mass (FM) and muscle mass (MM) [24]. Not all patients underwent BIA because several participants were pacemaker carriers; specifically, 2 patients from the LUX group and 2 patients from the SYN group.

Moreover, the waist and hip circumferences were measured and the waist to hip ratio was assessed, at all times of the study.

### 2.5. Laboratory Parameters

At T0, T1 and T2, we performed the following laboratory and urinary exams: azotemia, creatinine, UA, electrolytes, sideremia, glycaemia, lipid profile, chemical–physical urinary examination, albuminuria on morning urine. Moreover, inflammatory parameters such as C-reactive protein (CRP), erythrocyte sedimentation rate (ESR) and interleukin (IL)-6 were monitored at all time-points of the study. All patients also underwent capillary sampling using CR4000 tool for the oxidative stress evaluation and for the assessment of antioxidant defense mechanisms. In particular, a free oxygen radical test (FORT) and a free oxygen radical defense (FORD) test were performed [25,26]. The first test measures the levels of circulating oxygen free radicals [27] and the second indirectly determines the organisms’ antioxidant capacity by the concentration of ascorbic acid, glutathione, and albumin [28].

Laboratory parameters were monitored by Dimension Vista 1500 (Siemens Healthcare Diagnostics, Milano, Italy), while the lipid profile was detected by standard enzymatic colorimetric techniques (Roche Modular P800, Roche Diagnostics, Indianapolis, IN, USA) and IL-6 was monitored by IMMULITE 2000 XPi Immunoassay System (Siemens Healthcare Diagnostics, Milano, Italy).

All other parameters were analyzed according to standard procedures in the Clinical Chemical Laboratories of the University Hospital, PTV of Rome.

### 2.6. Statistical Analysis

All data were entered into an Excel spreadsheet (Microsoft, Redmond, WA, USA) and the analysis was performed using the Windows Social Science Statistics Package, version 25.0 (IBM_SPSS, Chicago, IL, USA).

The descriptive statistics consider the mean ± standard deviation for the parameters with normal distribution (after confirmation with histograms and the Kolgomorov–Smirnov test), while for the non-normal variables, they consider the median and the interval (minimum:maximum).

The comparison for the normal variables was carried out with a one-way ANOVA, while the comparisons between treatment over time (T0, T1 and T2) were conducted with a paired *t*-test for normal variables and a Mann–Whitney test for the non-normal variables. Regarding occurrences (percentages), the chi-square test, possibly corrected by Fisher’s exact test, was performed. A *p*-value < 0.05 was considered statistically significant.

## 3. Results

### 3.1. HPLC-DAD-MS: EVOOs Characterization

The two selected EVOOs were analyzed by HPLC-DAD-MS for evaluation of the MPCs content, the results of the analyses are reported in Table 1. Among the two EVOOs, monocultivar Mignolo (SYN) was the richest in phenolic compounds, with a value of 706.36 mg/kg compared to the blend of Tuscany cultivars (LUX) which presented a content of 485.01 mg/kg of total polyphenols.

Through the HPLC-DAD-MS analysis, it was possible to evaluate the content of single molecules. In particular, the values of hydroxytyrosol, tyrosol and derivatives were taken into consideration, as reported by the European Food Safety Authority (EFSA) EU No 432/2012 health claim. In fact, according to EFSA, it is necessary to consume 20 g/day of olive oil with a content of hydroxytyrosol and its derivatives of 5 mg, to have positive effects on health and to yield protection from oxidative stress [29].

The oleocanthal and oleacin content was evaluated in both EVOOs, given the importance of their antioxidant and anti-inflammatory effects, as described in 2005 by Beauchamp [30]. Mignolo (SYN) has an higher content of these molecules compared to LUX. In particular, SYN has a higher content of hydroxytyrosol at 70%, of oleacin and oleocanthal together representing 40%, and of oleuropein aglycone at 80%, in comparison to LUX. Regarding the content of MCPs, it is important to consider their bioavailability. In fact, the biodisponibility of hydroxytyrosol is dose-dependent and exerts physiological and systemic actions after its assumption through EVOO [31]. In particular, hydroxytyrosol is directly absorbed in the small intestine, while other phenolic compounds such as oleuropein aglycone are hydrolyzed, after EVOO consumption, in the small intestine to hydroxytyrosol [32]. In vitro study, investigating hydroxytyrosol stability in aqueous solution at various temperatures and concentrations, revealed that EVOOs with lower hydroxytyrosol concentrations were more susceptible to degradation. On the contrary, EVOOs that are higher in their content of hydroxytyrosol and derivatives were less prone to degradation processes. In addition, the studies available so far show that, in vivo, hydroxytyrosol did not undergo significant degradation processes after one week [33,34]. EVOO with a high content of MPCs stored in bottles, capped with nitrogen and made with special glass (that shielded 99.99% from UV radiation), after 18 months showed stable antioxidant and antiradical activities towards human low-density lipoprotein (LDL) oxidation. These beneficial actions are related to MPC content [35,36,37]. Currently, no human studies have been conducted to evaluate the bioavailability of hydroxytyrosol over time, however, recent studies have highlighted how hydroxytyrosol is more bioavailable in humans than in rats, both as a plasmatic and urinary metabolite [31,38]. Hydroxytyrosol is the molecule immediately available to carry out the antioxidant action of EVOO, while oleuropein aglycone and oleacin exert their antioxidant action by hydrolyzing and releasing hydroxytyrosol. Oleuropein aglycone and oleacin also display anti-inflammatory roles [15]. The synergistic and biodynamic cultivation of the olive trees of Mignolo (SYN) allowed us to obtain EVOO with a higher content of MPCs, compared to EVOO obtained from organic olive trees. Considering the results of the qualitative–quantitative characterization analyzes, the two EVOOs with different MPC contents were selected for application to patients in this study.

### 3.2. Study in Patients

A total of 40 CKD patients under conservative therapy were recruited for the in vivo study. The patients were divided into two subgroups, homogeneous for age, gender, and BMI. The first group assumed 40 mL/day of EVOO SYN, the second group assumed 40 mL/day of EVOO LUX, for 9 weeks. The epidemiological findings of the two study subgroups and their homogeneity are reported in the Table 2.

During the study, the patients dropped out due to factors unrelated to side effects deriving from EVOO consumption, as reported in Figure 1.

The laboratory and urinary parameters monitored at T0 (baseline), after 9 weeks of EVOO consumption (T1) and after 2 months of wash-out (T2) in the two study groups (SYN and LUX), are reported in Table 3. We observed a significant increase in the estimated glomerular filtration rate (e-GFR), calculated with the chronic kidney disease epidemiology collaboration (CKD-EPI) formula [39], after 9 weeks of SYN consumption (34.5 ± 16.8 mL/min/1.73 m^2^ vs. 37.7 ± 18.7 mL/min/1.73 m^2^; *p* = 0.0235) and this increase was maintained after the wash-out period (34.5 ± 16.8 mL/min/1.73 m^2^ vs. 38.2 ± 19.5 mL/min/1.73 m^2^; *p* = 0.0476), as reported in Table 4. In the SYN group, we also highlighted an albuminuria reduction (31.8 ± 45.2 mg/dL vs. 16.4 ± 24.1 mg/dL; *p* = 0.0413), an albumin enhancement (4.1 ± 0.3 g/dL vs. 4.3 ± 0.3 g/dL; *p* = 0.0413), an azotemia decrease (69.6 ± 23.1 mg/dL vs. 59.2 ± 17.3 mg/dL; *p* = 0.0346), a triglycerides reduction (154.5 ± 82.0 mg/dL vs. 132.7 ± 69.8 mg/dL; *p* = 0.0053) and a UA decrease (6.7 ± 1.5 mg/dL vs. 5.6 ± 1.2 mg/dL; *p* = 0.0206) at T1. After 2 months of wash-out, the increase in albumin (*p* = 0.0041), the reduction in azotemia (*p* = 0.0389) and the reduction in UA (*p* = 0.0230) were maintained over time, as reported in Table 4, while the reductions in triglyceride levels were not maintained over time (*p* = 0.0084). Moreover, we also demonstrated a reduction in inflammatory status, as monitored by CRP (4.2 ± 2.9 mg/L vs. 2.8 ± 2.3 mg/L; *p* = 0.0011), ERS (41.6 ± 24.7 mm/h vs. 35.1 ± 20.5 mm/h; *p* = 0.0005) and IL-6 (85.2 ± 110.7 pg/mL vs. 34.3 ± 11.1 pg/mL; *p* = 0.0321) at T1. Only the reduction in CRP was maintained after 2 months of wash-out (4.2 ± 2.9 mg/L vs. 2.7 ± 2.1 mg/L; *p* = 0.0007), while the ERS returned to basal levels at T2 (35.1 ± 20.5 mm/h vs. 43.9 ± 27.2 mm/h; *p* = 0.0218) and the IL-6 reduction was also maintained after the wash-out period (85.2 ± 110.7 vs. 42.8 ± 51.6, *p* = 0.043) (Table 5 and Table 6).

In the LUX group, we observed an albumin significant increase (4.2 ± 0.3 g/dL vs. 4.4 ± 0.4 g/dL; *p* = 0.0442), a significant UA reduction (6.6 ± 1.4 mg/dL vs. 6.0 ± 0.8 mg/dL; *p* = 0.0426) and an improvement in inflammatory status, highlighted by the reduction in CRP and ESR levels (respectively, 5.2 ± 4.5 mg/L vs. 3.0 ± 2.9 mg/L; *p* = 0.0070 and 38.5 ± 29.1 mm/h vs. 30.2 ± 24.4 mm/h; *p* = 0.0063). After 2 months of wash-out, we observed an increase in CRP (3.0 ± 2.9 mg/L vs. 4.89 ± 4.1 mg/L; *p* = 0.0072) and ESR (30.2 ± 24.4 mm/h vs. 36.7 ± 27.7 mm/h; *p* = 0.0090) levels and a decrease in serum albumin (4.4 ± 0.4 g/dL vs. 4.2 ± 0.3 g/dL; *p* = 0.0290) compared to T1.

The OS assessments of the two study subgroups are reported in Table 5. We showed a significant reduction in FORT values (289.3 ± 145.8 U vs. 194.0 ± 63.1 U; *p* = 0.00247) after 9 weeks of SYN consumption, but this reduction was not maintained after 2 months of wash-out, as reported in Table 6.

The body composition assessments of the two study subgroups are summarized in Table 7. We observed a significant decrease in reactance (39.3 ± 10.8 ohm vs. 42.3 ± 12.3 ohm; *p* = 0.0391), an TBW increase (52.8 ± 6.1% vs. 55.1 ± 5.9%; *p* = 0.0465), ICW enhancement (46.8 ± 7.2% vs. 49.9 ± 8.7%; *p* = 0.0058), a reduction in ECW (53.2 ± 7.2% vs. 50.1 ± 8.7%; *p* = 0.0058), an MM increase (38.3 ± 6.5% vs. 41.6 ± 7.2%; *p* = 0.0198) and a BCMI enhancement (9.1 ± 2.7 kg/m^2^ vs. 10.2 ± 3.6 kg/m^2^; *p* = 0.0230), after 9 weeks of SYN consumption. After 2 months of wash-out, we highlighted that the significant variation in reactance and MM, in respect to the baseline, were maintained after wash-out period (respectively, *p* = 0.0485 and *p* = 0.0400), as reported in Table 8. Instead, the BCMI values returned to baseline values (10.2 ± 3.6 kg/m^2^ vs. 9.1 ± 2.9 kg/m kg/m^2^; *p* = 0.0296).

In the LUX group, we detected a significant increase in TBW (56.3 ± 7.4% vs. 60.8 ± 8.3%; *p* = 0.0033) after 9 weeks of EVOO consumption, which decreased after 2 months of wash-out (60.8 ± 8.3% vs. 54.4 ± 7.4%; *p* = 0.0301).

## 4. Discussion

Consumption of EVOO MPCs seems to improve renal function biomarkers, in addition to the signs, and the metabolic dysfunctions related to CKD. Currently, in the literature the time scale of nutritional-intervention studies in CKD patients is variable and in most of them, this comprises between one and twenty-four weeks. For this reason, the choice of 9 weeks of treatment with EVOO and of 2 months of wash-out period has been driven by the mean time of treatment present in the literature [11,13,19,40,41,42,43,44].

**Figure 3:**
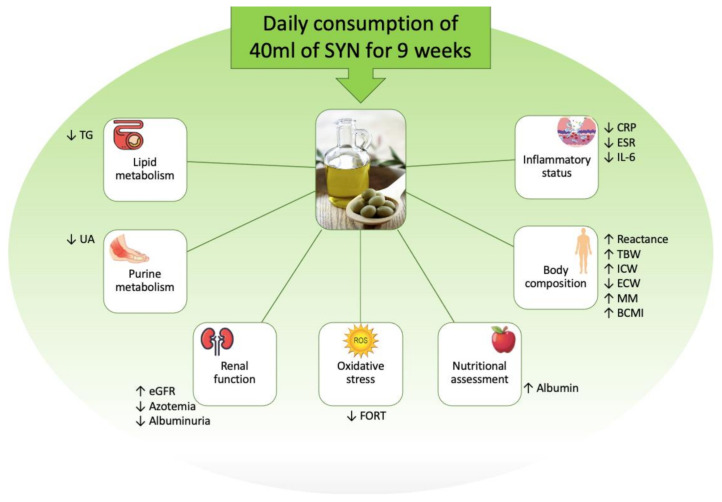
Outline of the study results. Abbreviations: BCMI, body cell mass index CKD, chronic kidney disease; CRP, C-reactive protein; ECW, extra cellular water; e-GFR, estimated glomerular filtration rate; ESR, erythrocyte sediment rate; FORT, Free Oxygen Radical Test; ICW, intracellular cell water; IL, interleukin; MM, muscle mass; TBW, total body water; TG, triglycerides; UA, uric acid.

In our population, in patients treated with SYN, we observed a significant improvement in e-GFR and a reduction in azotemia after 9 weeks of EVOO consumption, which persisted after the wash-out period (Figure 3). We hypothesize that the improvement in renal functional biomarkers was related to three mechanisms. The first, could be due to the reduction in OS, observed in the subgroup treated with SYN. In fact, as presented in the literature, OS can be found starting from the first stages of CKD [45,46] and is characterized by an accumulation of ROS and of advanced oxidation protein products (AOPPS), which cause direct damage to the podocyte and tubulointerstitial levels, at the same time inducing proteinuria [47,48]. The second mechanism could be related to the reduction in low-grade chronic inflammation [49], which is interconnected with the OS and plays a key role in the progression of nephropathy. The factors contributing to the development of the low-grade chronic inflammatory status in nephropathic patients, include increased production of inflammatory cytokines, OS itself, metabolic acidosis, intestinal dysbiosis, and altered metabolism of adipose tissue [50]. The third mechanism is related to the reduction in UA that is observed after SYN and LUX consumption. In fact, previous studies have shown that urate-lowering therapy is associated with slowing the progression of CKD [51,52].

Another important result obtained from our study, is the significant albuminuria reduction observed in the SYN subgroup. This amelioration is present not only at T1, but it also persists after the wash-out period (T2). The improvement in albuminuria is important in the clinical management of CKD patients as it is a well-known biomarker of kidney damage and it is one of the main factors associated with the progression of nephropathy. CKD progression seems to also be related to the activation of renal and systemic inflammatory pathways, which induces alterations of the glomerular filtration barrier, with consequent podocyte damage and development of proteinuria [53,54]. The improvement in albuminuria is also attributable to the observed reduction in OS and low-grade inflammatory status.

In our study, we observed a significant reduction in triglycerides in patients treated with SYN, as already highlighted by our preliminary data [55]. This effect seems to be dependent on the consumption of EVOO (SYN). In fact, after the wash-out period, we observed a return to basal values, probably due to CKD itself. Indeed, CKD, starting from the early stages, is characterized by an increase in triglyceride values [56,57]. The decrease in triglycerides levels could be explained by the high content of EVOO polyphenols and oleic acid and by their interaction with the various enzymes involved in energy metabolism [58,59].

Interestingly, in the two subgroups we also observed a decrease in UA values at T1, compared to T0. In detail, patients treated with SYN showed a statistically significant reduction in UA values, after 9 weeks of EVOO consumption, which lasted until the time-point T2. While, in the LUX subgroup, although a statistically significant reduction was observed at T1, this was limited to the period of EVOO intake. In fact, after the wash-out period, the UA concentration increased up to baseline values.

CKD is associated with an increase in UA values due both to its reduced renal excretion and to its altered tubular transport in the nephron [60]. The UA increase observed in CKD patients seems to favor OS through the inhibition of nicotinamide adenine dinucleotide phosphate (NADPH) oxidase, altering the vascular response to nitric oxide [61]. Furthermore, it seems to exert a pro-inflammatory action by activating transcription of the factor nuclear factor kappa-light-chain-enhancer of activated B cells (NF-kB) [62] and stimulating the synthesis of tumor necrosis factor (TNF)-α and IL-1 [63]. We hypothesized that the UA reduction observed in our patients after 9 weeks of SYN consumption, may be due to two probable mechanisms. The first one involves the intestinal excretion pathway of UA. In fact, the intake of the polyphenols present in the EVOO, may have positively modulated the gut microbiota composition and increased its intestinal excretion, as previously highlighted in the literature [64,65,66,67,68]. The second mechanism could be related to the enhancement of energy metabolism induced by the consumption of SYN, as evidenced by our impedance data, related to basal metabolism and by a previous study [69]. This would lead to a lower availability of adenosine monophosphate (AMP), a UA precursor. The prolonged effect over time is probably due to two mechanisms. The first one is related to the peculiar bioavailability of the antioxidant molecules present in the administered EVOOs. In fact, these molecules, such as oleuropein aglycone and oleacin, represent a reserve of hydroxytyrosol, which is released after hydrolysis reactions. This effect was highlighted by Hu et al. in 2014, in which, the metabolic pathway of hydroxytyrosol—resulting from the hydrolysis of oleuropein aglycone—secondary to consumption of EVOO consumption, was described. The authors showed a slow release and prolonged antioxidant activity of EVOO [70]. Similarly, considering the structure of oleacin—a secoiridoide in presence of hydroxytyrosol—it is hypothesized that the pharmacokinetics described for oleuropein aglycone in EVOO can also be expected for both molecules. Namely, the oleacin shows a prolonged antioxidant activity after consumption of EVOO [70]. This first mechanism may allow for the Mignolo SYN—with a high content of oleacin and oleuropein aglycone—to exert a prolonged antioxidant effect, compared to the EVOO LUX. The second mechanism is related to the impact of EVOO’s polyphenols on the gut microbiota composition. Previous studies have demonstrated that EVOO’s polyphenols are able to modulate gut microbiota and the production of short chain fatty acids (SCFAs). In fact, some studies have demonstrated that EVOO’s polyphenols induce increased production of butyrate or gut bacteria producing butyrate, enhancing the α-divesity of gut micriobiota, which induces beneficial systemic effects that seem to be maintained over the time [68,69].

In our study, we confirmed the anti-inflammatory action of EVOO MCPs [14,15,70]. In fact, our patients after SYN and LUX consumption, showed a significant reduction in both CRP and ESR. Specifically, this anti-inflammatory effect in SYN patients tended to persist over time, as evidenced by the CRP improvement, which was maintained after the wash-out period, while in LUX patients this effect was limited to the period of EVOO consumption. Moreover, in SYN patients, we observed a significant IL-6 reduction after 9 weeks of EVOO consumption, and at T2. The higher presence of oleocanthal in SYN would explain the observed anti-inflammatory effects. In fact, oleocanthal inhibits cyclooxygenase (COX)-1 and COX-2 enzymes in a dose-dependent manner, its anti-inflammatory action seems to be similar to the well-known non-steroidal anti-inflammatory drug, ibuprofen. Beauchamp and collaborators showed that the inhibitory action of oleocanthal on COX-1 and COX-2 enzymes, is greater than that induced by ibuprofen at the same concentration [30]. In our study, the anti-inflammatory action of oleocanthal was preserved by consumption of raw EVOO. In fact, previous studies have shown how cooking reduces the biological activities of oleocanthal [71]. EVOO anti-inflammatory action is due to also the inhibition of TNF-α and IL-4 synthesis, as demonstrated in a cell line derived from human blood (KU812 cells) [72,73].

The observed increase in albumin values, noted in both treated subgroups at T1, is in agreement with previous studies [74,75] and it seems to be associated with reductions in the chronic inflammatory status. Previous data have highlighted how the inflammatory state reduces the synthesis of serum albumin and that its concentration is inversely correlated to that of acute phase proteins [76]. This significant albumin increase in the SYN subgroup persists after wash-out, while the albumin value returns to baseline in the LUX subgroup.

Furthermore, only in patients who consumed SYN, we observed a significant reduction in OS, monitored by FORT. These data do not seem to last over time, as OS tended to rise again 2 months after the wash-out period, yet remaining lower than the baseline values. The antioxidant action is exerted by the EVOO polyphenols, as suggested by previous studies [77,78]. In particular, polyphenols inhibit the enzymes involved in the formation of ROS, exert a scavenger action against ROS, and upregulate the antioxidant defense mechanisms [79,80]. The synergistic antioxidant effect of the MPCs present in high quantities in SYN, is greater in SYN in respect to LUX.

As regards the body composition assessment—monitored by BIA—we, interestingly, observed a BCMI improvement in SYN patients at T1. In the literature, it has been highlighted that BCMI is a very sensitive index for monitoring MM changes, which, in our study population, may be related to uremic sarcopenia [81,82,83]. BCMI seems to be a prognostic index of nutritional, inflammatory, and muscle mass status, as previously observed in geriatric patients [84]. In our study, in addition to the improvement in BCMI, we also observed an increase in MM% in the SYN subgroup at T1 and after the wash-out period (T2). The increase in the two parameters seems to be related to each other and associated with the improvement in the inflammatory state observed.

In addition, we detected an increase in reactance in the SYN subgroup; a parameter related to the cellular integrity and nutritional status of the patients. These data agree with the albumin increase highlighted in the study. In fact, both parameters express an improvement in the patient’s nutritional status [85].

Finally, we demonstrated a TBW% increase in both subgroups (SYN and LUX) at T1 and, at the same time, an improvement in the ICW% to ECW% ratio only in the SYN subgroup. These parameters agree with the observed MM% increase, which is characterized by a high percentage of ICW [86].

## 5. Conclusions

Our study shows that daily use of an EVOO with high MPC content seems to exert an important anti-inflammatory and antioxidant action in nephropathic patients. Therefore, once again, the pivotal role that nutritional-diet therapy plays in the clinical management of CKD patients and the consequential improvements in their quality of life, exerting positive effects on CKD signs and symptoms, has been highlighted. Thus, this study in nephropathic patients may lay the foundations for further randomized clinical trials that confirm the important role played by EVOO MPCs in the management of CKD.

## Figures and Tables

**Table 1:** High-performance liquid chromatography (HPLC-DAD-MS) analysis of EVOOs samples.

Compound	SYN	LUX
Hydroxytyrosol (mg/L *)	1.78 ± 0.05	0.5 ± 0.02
Tyrosol (mg/L *)	1.71 ± 0.05	0.5 ± 0.03
Elenolic acid derivatives (mg/L *)	198.76 ± 5.96	32.26 ± 0.97
Elenolic acid (mg/L *)	28.15 ± 0.84	129.1 ± 3.87
Oleacin (mg/L *)	121.98 ± 3.66	77.54 ± 2.33
Oleocanthal (mg/L *)	46.02 ± 1.38	41.23 ± 1.24
Oleuropein aglycone (mg/L *)	142.65 ± 4.28	23.95 ± 0.72
Secoiridoids derivatives (mg/L *)	34.29 ± 1.03	87.47 ± 2.62
Lignans (mg/L*)	131.02 ± 3.93	92.44 ± 2.77
Total MCP (mg/L*)	706.36 ± 21.19	485.01 ± 14.55

* All results are the average of three determinations and the standard error is <2.5%. Abbreviations: LUX, Luxolio; SYN, Synergy.

**Table 2:** Clinical features of the study populations.

	SYN	LUX	*p* (ANOVA Test)
N	20	20	
Gender (male/female)	13/7	12/8	ns
Age (years)	70.6 ± 12.4	68.5 ± 11.4	ns
BMI (kg/m^2^)	28.9 ± 3.7	27.5 ± 5.9	ns

All data are expressed as mean ± standard deviation; abbreviations: ns, not significant; BMI, Body mass index; LUX, Luxolio; SYN, Synergy.

**Table 3:** Laboratory and urinary parameters of two groups of the study.

	SYN					LUX				
	T0	T1	*p*-Value (T0 vs. T1)	T2	*p*-Value (T1 vs. T2)	T0	T1	*p*-Value (T0 vs. T1)	T2	*p*-Value (T1 vs. T2)
Creatinine (mg/dL)	2.1 ± 0.8	2.0 ± 0.9	ns ^#^	2.1 ± 1.0	ns ^#^	2.2 ± 1.2	2.1 ± 1.2	ns ^#^	2.2 ± 1.2	ns ^#^
e-GFR (mL/min/1.73 m^2^)	34.5 ± 16.8	37.7 ± 18.7	0.0235 ^#^	38.2 ± 19.5	ns ^#^	37.7 ± 20.0	40.2 ± 21.0	ns ^#^	37.5 ± 20.0	ns ^#^
Albuminuria (mg/dL)	31.8 ± 45.2	16.4 ± 24.1	0.0413 ^#^	28.8 ± 45.1	ns ^#^	30.8 ± 59.6	18.8 ± 41.13	ns ^#^	22.7 ± 55.2	ns ^#^
Albumin (g/dL)	4.1 ± 0.3	4.3 ± 0.3	0.0039 ^#^	4.3 ± 0.3	ns ^#^	4.2 ± 0.3	4.4 ± 0.4	0.0442 ^#^	4.2 ± 0.3	0.0290 ^#^
Azotaemia (mg/dL)	69.6 ± 23.1	59.2 ± 17.3	0.0346 ^#^	61.3 ± 17.6	ns ^#^	65.4 ± 18.8	60.3 ± 22.0	ns ^#^	63.7 ± 21.9	ns ^#^
Sodium (mEq/L)	139.7 ± 3.4	139.5 ± 2.5	ns ^#^	139.6 ± 2.6	ns ^#^	141.6 ± 1.9	140.1 ± 2.5	ns ^#^	141.2 ± 1.8	ns ^#^
Potassium (mEq/L)	4.4 ± 0.6	4.4 ± 0.6	ns ^#^	4.3 ± 0.5	ns ^#^	4.5 ± 0.4	4.4 ± 0.4	ns ^#^	4.5 ± 0.5	ns ^#^
Calcium (mg/dL)	9.8 ± 0.5	9.6 ± 0.4	ns ^#^	9.6 ± 0.4	ns ^#^	9.8 ± 0.4	9.6 ± 0.5	ns ^#^	9.7 ± 0.4	ns ^#^
Phosphorus (mg/dL)	3.4 ± 0.6	3.3 ± 0.6	ns ^#^	3.3 ± 0.6	ns ^#^	3.6 ± 0.6	3.5 ± 0.6	ns ^#^	3.7 ± 0.7	ns ^#^
TC (mg/dL)	175.1 ± 44.9	169.5 ± 37.5	ns ^#^	147.7 ± 47.2	ns ^#^	178.9 ± 56.1	181.0 ± 47.7	ns ^#^	180.9 ± 52.6	ns ^#^
HDL-C (mg/dL)	41.8 ± 13.3	41.3 ± 12.7	ns ^#^	42.4 ± 11.7	ns ^#^	44.5 ± 12.3	47.3 ± 12.3	ns ^#^	46.0 ± 11.5	ns ^#^
LDL-C (mg/dL)	100.6 ± 30.8	100.0 ± 29.2	ns ^#^	102.1 ± 21.6	ns ^#^	112.1 ± 47.1	103.6 ± 39.8	ns ^#^	112.5 ± 45.7	ns ^#^
Triglycerides (mg/dL)	154.5 ± 82.0	132.7 ± 69.8	0.0053 ^#^	147.0 ± 79.9	0.0084 ^#^	123.4 ± 63.7	120.4 ± 55.2	ns ^#^	128.2 ± 55.2	ns ^#^
Sideremia (μg/dL)	86.8 ± 34.2	73.6 ± 18.1	ns ^#^	73.9 ± 17.6	ns ^#^	75.5 ± 22.4	68.6 ± 20.4	ns ^#^	75.0 ± 21.5	ns ^#^
Glycaemia (mg/dL)	98.5 ± 25.3	96.7 ± 35.4	ns ^#^	98.7 ± 37.3	ns ^#^	93.4 ± 19.8	89.8 ± 13.2	ns ^#^	89.2 ± 17.6	ns ^#^
Uric acid (mg/dL)	6.7 ± 1.5	5.6 ± 1.2	0.0206 ^#^	6.1 ± 1.6	ns ^#^	6.6 ± 1.4	6.0 ± 0.8	0.0426 ^#^	6.3 ± 1.6	ns ^#^
CRP (mg/L)	4.2 ± 2.9	2.8 ± 2.3	0.0011 ^#^	2.7 ± 2.1	ns ^#^	5.2 ± 4.5	3.0 ± 2.9	0.0070 ^#^	4.89 ± 4.1	0.0072 ^#^
ESR (mm/h)	41.6 ± 24.7	35.1 ± 20.5	0.0005 ^#^	43.9 ± 27.2	0.0218 ^#^	38.5 ± 29.1	30.2 ± 24.4	0.0063 ^#^	36.7 ± 27.7	0.0090 ^#^

All data are expressed as mean ± standard deviation; ^#^ for the comparison of each parameter we applied test: *t*-test for paired data. Values of *p* ≤ 0.05 are considered statistically significant. Abbreviations: LUX, Luxolio; SYN, Synergy; e-GFR, estimated glomerular filtration rate; TC, total-cholesterol; HDL-C, high-density lipoprotein cholesterol; LDL-C, low-density lipoprotein cholesterol; ESR, erythrocyte sedimentation rate; ns, not significant; CRP, C-reactive protein.

**Table 4:** *p*-values of baseline vs. wash-out period of laboratory and urinary parameters.

	SYN	LUX
	*p*-Value (T0 vs. T2)	*p*-Value (T0 vs. T2)
Creatinine (mg/dL)	ns ^#^	ns ^#^
e-GFR (mL/min/1.73 m^2^)	0.0476 ^#^	ns ^#^
Albuminuria (mg/dL)	ns ^#^	ns ^#^
Albumin (g/dL)	0.0041 ^#^	ns ^#^
Azotaemia (mg/dL)	0.0389 ^#^	ns ^#^
Sodium (mEq/L)	ns ^#^	ns ^#^
Potassium (mEq/L)	ns ^#^	ns ^#^
Calcium (mg/dL)	ns ^#^	ns ^#^
Phosphorus (mg/dL)	ns ^#^	ns ^#^
TC (mg/dL)	ns ^#^	ns ^#^
HDL-C (mg/dL)	ns ^#^	ns ^#^
LDL-C (mg/dL)	ns ^#^	ns ^#^
Triglycerides (mg/dL)	ns ^#^	ns ^#^
Sideremia (μg/dL)	ns ^#^	ns ^#^
Glycaemia (mg/dL)	ns ^#^	ns ^#^
Uric acid (mg/dL)	0.0230 ^#^	ns ^#^
CRP (mg/L)	0.0007 ^#^	ns ^#^
ESR (mm/h)	ns ^#^	ns ^#^

^#^ Applied test: *t*-test for paired data. Values of *p* ≤ 0.05 are considered statistically significant. Abbreviations: LUX, Luxolio; SYN, Synergy; e-GFR, estimated glomerular filtration rate; TC, total-cholesterol; HDL-C, high-density lipoprotein cholesterol; LDL-C, low-density lipoprotein cholesterol; CRP, C-reactive protein; ESR, erythrocyte sedimentation rate; ns, not significant.

**Table 5:** Oxidative stress assessment of two groups of the study.

	SYN					LUX				
	T0	T1	*p*-Value (T0 vs. T1)	T2	*p*-Value (T1 vs. T2)	T0	T1	*p*-Value (T0 vs. T1)	T2	*p*-Value (T1 vs. T2)
FORT (U)	289.3 ± 145.8	194.0 ± 63.1	0.00247 ^#^	223.3 ± 87.9	ns ^#^	352.4 ± 156.5	278.6 ± 128.2	ns ^#^	337.2 ± 143.7	ns ^#^
FORD (mmol/L Trolox)	1.3 ± 0.4	1.2 ± 0.5	ns ^#^	1.1 ± 0.4	ns ^#^	1.3 ± 0.5	0.9 ± 0.5	ns ^#^	1.5 ± 0.6	ns ^#^
IL-6 (pg/mL)	85.2 ± 110.7	34.3 ± 11.1	0.0321 ^#^	42.8 ± 51.6	ns ^#^	77.9 ± 98.3	59.8 ± 25.7	ns ^#^	65.2 ± 34.2	ns ^#^

All data are expressed as mean ± standard deviation; ^#^ for the comparison of each parameter we applied test: *t*-test for paired data. Values of *p* ≤ 0.05 are considered statistically significant. Abbreviations: IL, interleukin; LUX, Luxolio; SYN, Synergy; ns, not significant; FORT, free oxygen radical test; FORD, free oxygen radical defense.

**Table 6:** *p*-value of baseline vs. wash-out period of oxidative stress assessment.

	SYN	LUX
	*p*-Value (T0 vs. T2)	*p*-Value (T0 vs. T2)
FORT (U)	ns ^#^	ns ^#^
FORD (mmol/L Trolox)	ns ^#^	ns ^#^
IL-6 (pg/mL)	0.043 ^#^	ns ^#^

^#^ Applied test: *t*-test for paired data. Values of *p* ≤ 0.05 are considered statistically significant. Abbreviations: IL, interleukin; LUX, Luxolio; SYN, Synergy; ns, not significant; FORT, Free Oxygen Radical Test; FORD, Free Oxygen Radical Defense.

**Table 7:** Body composition assessment of two groups of the study.

	SYN					LUX				
	T0	T1	*p*-Value (T0 vs. T1)	T2	*p*-Value (T1 vs. T2)	T0	T1	*p*-Value (T0 vs. T1)	T2	*p*-Value (T1 vs. T2)
Weight (kg)	79.1 ± 14.1	78.8 ± 13.8	ns ^#^	78.4 ± 13.2	ns ^#^	75.8 ± 15.8	75.6 ± 15.5	ns ^#^	76.6 ± 15.0	ns ^#^
BMI (kg/m^2^)	28.9 ± 3.7	28.8 ± 3.4	ns ^#^	28.7 ± 3.7	ns ^#^	27.5 ± 5.9	27.4 ± 5.8	ns ^#^	27.8 ± 5.8	ns ^#^
Waist to hip ratio	0.93 ± 0.07	0.92 ± 0.07	ns ^#^	0.92 ± 0.06	ns ^#^	0.96 ± 0.09	0.95 ± 0.09	ns ^#^	0.95 ± 0.8	ns ^#^
Resistance (ohm)	480.4 ± 87.3	450.8 ± 79.1	ns ^#^	472.8 ± 96.9	ns ^#^	498.7 ± 81.0	486.2 ± 98.2	ns ^#^	496.0 ± 74.5	ns ^#^
Reactance (ohm)	39.3 ± 10.8	42.3 ± 12.3	0.0391 ^#^	41.9 ± 11.3	ns ^#^	41.8 ± 12.1	40.2 ± 10.0	ns ^#^	41.8 ± 8.7	ns ^#^
Phase angle (°)	4.6 ± 1.5	5.3 ± 2.2	ns ^#^	4.8 ± 1.5	ns ^#^	4.9 ± 0.5	5.4 ± 0.7	ns ^#^	4.9 ± 0.5	ns ^#^
TBW (%)	52.8 ± 6.1	55.1 ± 5.9	0.0465 ^#^	53.2 ± 6.3	ns ^#^	56.3 ± 7.4	60.8 ± 8.3	0.0033 ^#^	54.4 ± 7.4	0.0301 ^#^
ICW (%)	46.8 ± 7.2	49.9 ± 8.7	0.0058 ^#^	47.0 ± 9.0	ns ^#^	48.5 ± 5.0	49.5 ± 6.3	ns ^#^	45.1 ± 14.4	ns ^#^
ECW (%)	53.2 ± 7.2	50.1 ± 8.7	0.0058 ^#^	53.0 ± 8.8	ns ^#^	51.5 ± 3.1	50.5 ± 3.5	ns ^#^	54.9 ± 3.0	ns ^#^
FM (%)	34.4 ± 8.2	31.5 ± 8.2	ns ^#^	32.6 ± 8.0	ns ^#^	28.6 ± 9.6	22.2 ± 10.1	ns ^#^	26.5 ± 8.5	ns ^#^
FFM (%)	65.6 ± 7.6	68.5 ± 8.2	ns ^#^	67.4 ± 7.9	ns ^#^	71.4 ± 9.6	77.8 ± 10.5	ns ^#^	73.5 ± 9.9	ns ^#^
MM (%)	38.3 ± 6.5	41.6 ± 7.2	0.0198 ^#^	40.7 ± 7.5	ns ^#^	43.9 ± 6.7	45.5 ± 5.9	ns ^#^	44.3 ± 706	ns ^#^
BCMI (kg/m^2^)	9.1 ± 2.7	10.2 ± 3.6	0.0230 ^#^	9.1 ± 2.9	0.0296 ^#^	8.9 ± 1.7	9.7 ± 2.3	ns ^#^	8.3 ± 2.9	ns ^#^

All data are expressed as mean ± standard deviation; ^#^ for the comparison of each parameter we applied test: *t*-test for paired data; values of *p* ≤ 0.05 are considered statistically significant. Abbreviations: LUX, Luxolio; SYN, Synergy; BMI, body mass index; TBW, total body water; ICW, intra cell water; ECW, extra cell water, FM, fat mass; FFM, fat free mass; MM, muscle mass; BCMI, body cellular mass index.

**Table 8:** *p*-value of baseline vs. wash-out period of body composition assessment.

	SYN	LUX
	*p*-Value (T0 vs. T2)	*p*-Value (T0 vs. T2)
Weight (kg)	ns ^#^	ns ^#^
BMI (kg/m^2^)	ns ^#^	ns ^#^
Waist/hip ratio	ns ^#^	ns ^#^
Resistance (ohm)	ns ^#^	ns ^#^
Reactance (ohm)	0.0485 ^#^	ns ^#^
Phase angle (°)	ns ^#^	ns ^#^
TBW (%)	ns ^#^	ns ^#^
ICW (%)	ns ^#^	ns ^#^
ECW (%)	ns ^#^	ns ^#^
FM (%)	ns ^#^	ns ^#^
FFM (%)	ns ^#^	ns ^#^
MM (%)	0.0400 ^#^	ns ^#^
BCMI (kg/m^2^)	ns ^#^	ns ^#^

^#^ For the comparison of each parameter we applied test: *t*-test for paired data; values of *p* ≤ 0.05 are considered statistically significant. Abbreviations: LUX, Luxolio; SYN, Synergy; BMI, body mass index; TBW, total body water; ICW, intra cell water; ECW, extra cell water, FM, fat mass; FFM, fat free mass; MM, muscle mass; BCMI, body cellular mass index.

## Data Availability

Data available on request due to privacy restrictions. The data presented in this study are available on request from the corresponding author.
